# Biometric Image Analysis for Quantitation of Dividing Platelets

**DOI:** 10.3390/mi10010001

**Published:** 2018-12-20

**Authors:** Hyun-Jeong Kim, Yejin Song, Jaewoo Song

**Affiliations:** Department of Laboratory Medicine, Yonsei University College of Medicine, Yonsei-ro 50-1, Seodaemun-gu, Seoul 03722, Korea; HJKIM0530@yuhs.ac (H.-J.K.); chaos0581@naver.com (Y.S.)

**Keywords:** platelet, division, differential doublet counting

## Abstract

(1) Background: Quantification of platelet division is challenging because automated Coulter cell counters produce equivocal platelet counts. (2) Methods: We applied the flow cytometric cell tracking dye dilution assay as a popular immunological method to evaluate lymphocyte proliferation to prove and quantitate platelet division. We also devised a method relying on platelet culture in a semisolid medium which enabled dividing platelets to be identified by limiting the diffusive movement of platelets. Mixing platelets of different labeling colors in semisolid medium and counting the platelet doublets of each color combination enabled us to prove and quantitate platelet division. (3) Results: The tracking dye dilution assay revealed that 75.5 to 85.6% of platelets were dividing after 20 hours in culture. Platelets labeled with two different tracking dyes were mixed and cultured in semisolid medium for differential doublet counting. We counted platelet singlets and doublets of each color and color combination using confocal microscopy after six hours of culture and compared the relative number of two-colored doublets with binomial prediction to prove platelet division (*P* < 0.01). Division was suppressed by taxol, nocodazole, or cytochalasin D treatment. We derived a formula for determining the fraction of dividing platelets using the numbers of singlets and doublets of each color and color combination. The platelet division fraction ranged from 8.8 to 17.5%. (4) Conclusion: We successfully measured platelet division using a simple biometric image analysis method with possible future application to microfluidic devices.

## 1. Introduction

### 1.1. Background

Platelets are quasi-cellular particulate blood components circulating in blood vessels. Similar to red blood cells, platelets lack nuclei and are unable to actively express genes embedded in DNA strands, but their biological roles, both physiologic and pathologic, are enormous [1,2]. The most important and well-known function of platelets is hemostasis, or prevention of blood loss at sites of tissue injury. Platelets function as the first-line defense mechanism against blood loss by promptly aggregating around vascular breakages. These platelet aggregates are often called “the primary hemostatic plug” because this structure clogs the hole in a blood vessel in a pure physical sense. For the hemostatic plug to be effective, it must form a mass of a certain size, and this is one of the reasons why the platelet count is important. Furthermore, platelets carry out two preparatory cellular actions, adhesion and secretion, in the process of aggregation [1,3,4]. Extravasated platelets first attach to the perivascular tissue through numerous receptors on their surface, the most crucial of which are the collagen receptors. The attached platelets are activated internally through the action of those receptors and intracellular signal molecules to begin producing and secreting active substances. These secreted substances are important for inducing the activation of inert platelets passing by and recruiting them to the growing hemostatic plug. If there are few attached platelets in the beginning, then the growth of the hemostatic plug is significantly hampered.

### 1.2. The Problem of Low Platelet Count

The platelet count decreases below the critical limit in various diseases and clinical conditions. Below a platelet count of 5 × 10^10^/L, there is a high risk of excessive bleeding during invasive medical or surgical procedures, necessitating preemptive transfusion to raise the platelet count [5,6,7]. Below 1 × 10^10^/L, the risk of spontaneous bleeding rises significantly [7]. The most well-known disease featuring a decreased platelet count is idiopathic thrombocytopenic purpura, a kind of autoimmune disorder where the immune reaction is spuriously directed toward the patient’s own platelets [8]. In a subset of the patients, the platelet count does not recover spontaneously, and the disease progresses to the chronic phase [9]. For these patients, many therapeutic options can be considered, ranging from corticosteroid treatment to surgical removal of the spleen [10]. Recently, chemical therapeutics that mimic the action of natural hematopoietic substances promoting platelet production were introduced to manage refractory patients [11,12]. However, even the latest treatment modality is imperfect, and undesirable side effects, including bone marrow fibrosis, have been reported [13]. In addition to idiopathic thrombocytopenia, there are many other clinical conditions associated with a decreased platelet count, though less well-recognized. 

### 1.3. Platelet Division

Platelet division in peripheral blood as a means of increasing the platelet count can be meaningful. Unlike dividing cells, platelets are generally regarded as unproductive in the sense that they are anuclear fragments of another cell, the megakaryocyte, and incapable of the canonical cycle of cell division [14]. However, recent studies presented evidence that platelets in peripheral blood could form progeny or at least undergo fission. Schwertz, et al. demonstrated the existence of intermediate forms of dividing platelets in platelet culture medium. By analyzing electron microscopy images and observing single platelets contained in isolated medium droplets, they found vivid evidence that platelets do divide with their size and intracellular contents preserved [15]. Thon, et al. isolated large platelets freshly released from bone marrow (the hematopoietic tissue) by density gradient centrifugation [16]. They observed the generation of new, smaller platelets from those large platelets, with a clear increase in platelet count after culture. These observations indicate the existence of a post-hematopoietic mechanism influencing platelet count in peripheral blood. However, demonstrating platelet division is one matter, while analyzing how many platelets divide and at what rate is another. The quantitative assessment of platelet division is difficult because of the lack of proper tools. Automated Coulter cell counters often produce equivocal platelet counts. They poorly differentiate single platelets from doublets, triplets, or other small-sized clumps. Manual chamber counting is cumbersome, and the visual distinction of single platelets from every small clump on a large scale is impractical. Here, we introduce simple methods for the quantitative assessment of platelet division in in-vitro culture conditions, with potential future applications for microfluidic devices.

## 2. Materials and Methods 

Healthy adult volunteers were recruited for blood collection with written informed consent. The study was approved by the Institutional Review Board of Yonsei University College of Medicine and performed in accordance with the Declaration of Helsinki.

### 2.1. Platelet Isolation from Blood

Blood from healthy donors was drawn into tubes containing an acid citrate dextrose (ACD) anticoagulant. Platelet rich plasma (PRP) was obtained by centrifugation at 250g for 15 minutes. Platelets were pelleted by centrifugation of the PRP at 2200g for 15 minutes and washed twice in Tyrode’s buffer (10 mM HEPES, 135 mM NaCl, 2.8 mM KCl, 1 mM MgCl_2_, 2 mM CaCl_2_, 12 mM NaHCO_3_, 0.4 mM NaOH_2_PO_4_, and 5.5 mM Glucose, pH 7.4) in the presence of 1 μM prostaglandin I_2_ (Sigma Aldrich, St. Louis, MO, USA). Finally, platelets were resuspended in Tyrode’s buffer with 0.35% human serum albumin (SK plasma, Seongnam, Korea), and the platelet count was measured using an automated hematology analyzer (Coulter counter; Advia2120i, Siemens, Munich, Germany) adjusted to meet the purpose of each experiment.

### 2.2. Platelet Count before and after Suspension Culture Measured Using an Automated Coulter Counter

We resuspended the washed platelets in M199 (ThermoFisher Scientific, Waltham, MA, USA) cell culture medium without added substances. We used a rotator designed to be used in a cell culture incubator (MacsMix, Miltenyi Biotec, Bergisch Gladbach, Germany) to prevent platelet sedimentation during the suspension culture. Tubes containing the platelet suspension were placed properly in the adaptor of the rotator and set to gentle rotation. The rotator and the culture tubes were then put into a cell culture incubator for six hours. Before starting a culture, an aliquot of platelet suspension in liquid medium was collected, and the platelet count was measured in triplicate using the automated hematology analyzer (Coulter counter, Advia 2120i, Siemens). After six hours of culture, platelet counts were measured in triplicate and compared to the initial count.

### 2.3. CFSE Dilution Assay to Assess Platelet Division

#### 2.3.1. Principle

**Figure 1:**
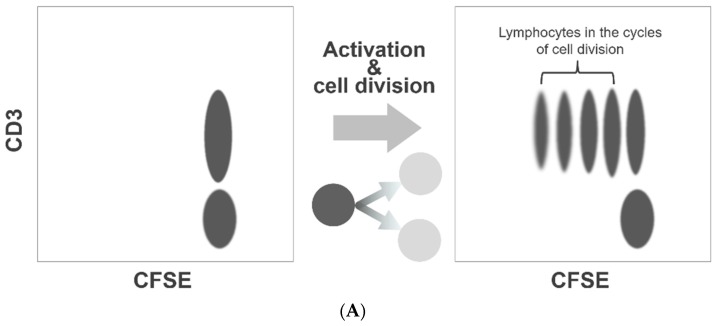

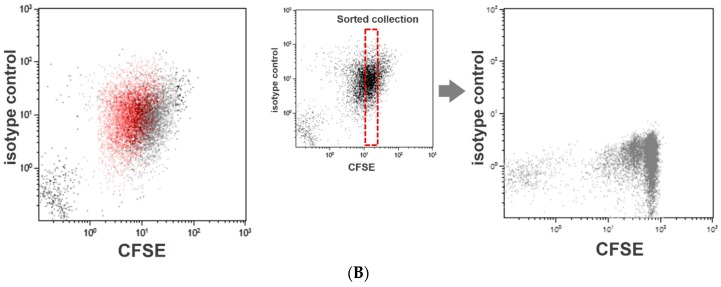
CFSE dilution assay for platelet division. Flow cytometry-based assessment of platelet division is based on the method to quantitate lymphocyte activation/proliferation, known as the CFSE dilution assay. As the labeled lymphocytes divide and proliferate, the labeling dye dilutes to form separate clusters with a weaker fluorescence intensity (**A**). We used this assay to examine platelet division. However, the fluorescence intensity of labeled platelets varied widely, and the clusters with decreased fluorescence shift could not be separated from the original platelet cluster. To enable a clear distinction of dividing platelets with reduced fluorescence, we conducted flow cytometric sorting to select platelets with a narrow range of fluorescence intensity (**B**).

Our method of the flow cytometric assessment of platelet division is based on the method that was originally developed to quantitate lymphocyte activation and proliferation, the carboxyfluorescein diacetate N-succinimidyl ester (CFSE) dilution assay. This is a popular and widely practiced procedure in immunology research [17,18]. The CFSE-labeled lymphocytes form a cluster of dots on a relevant fluorescence dot plot (Figure 1A). As the labeled lymphocytes divide, the dye is diluted to less than a half of the original amount per cell. Consequently, on the dot plot presentation, the fluorescence intensities of the lymphocytes undergoing cell division decrease discretely to form separate clusters on the left side of the undivided lymphocyte cluster (Figure 1A). We applied this method to assess platelet division. However, unlike labeled lymphocytes, the fluorescence intensity of labeled platelets varied widely, and the clusters with shifted fluorescence intensity could not be separated from the original platelet cluster (Figure 1B). To overcome this limitation, we resorted to a flow cytometric cell sorting technique. By applying a narrow sorting gate for CFSE fluorescence, we selectively collected platelets showing a very narrow range of CFSE fluorescence intensity. Practically, this method of sorting can be compared to squeezing labeled platelets through a narrow fluorescence intensity slit. With the limited dispersion of CFSE fluorescence, a discrete dot cluster with a small shift in fluorescence intensity could be detected and quantified.

#### 2.3.2. Procedure for Assessment of Platelet Division

CFSE (Sigma Aldrich) was added to the buffered platelet suspension to a final concentration of 1.0 μM, followed by incubation for five minutes at room temperature. After incubation, the unincorporated dye was removed by washing with Tyrode’s buffer. Then, the labeled platelets were resuspended in Tyrode’s albumin buffer. The sorting of the CFSE-labeled platelets was conducted by FACSAria™ III (BD Biosciences, San Jose, CA, USA). Platelet singlets were first selected for analysis and collection, and at least 2 × 10^7^ platelets were collected per tube filled with M199 medium. Each round of the sorting procedure was completed within an hour. The collected platelet suspension in M199 medium was cultured in the cell culture incubator for 20 hours. Aliquots of platelet suspension were collected for flow cytometry analysis (LSR2, BD Biosciences) before culture and after the sixth hour of culture. After 20 hours of culture, the platelets in the medium were again analyzed by flow cytometry. The generation of new discrete clusters of CFSE fluorescence and the size of each cluster were examined on dot plots and histograms.

### 2.4. Differential Counting of Platelet Doublets

#### 2.4.1. Principle

**Figure 2:**
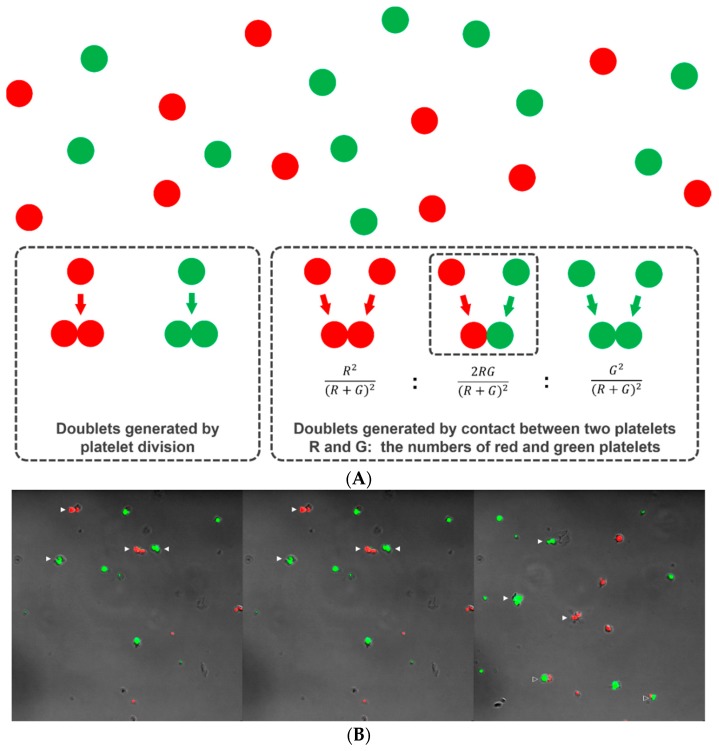
Platelet doublets developing from division and incidental contact. Two platelet suspensions were separately labeled with CFSE (green) and Deep Red (red), mixed together in a viscous semisolid medium and cultured for six hours. Platelet doublets can form when two platelets come into incidental contact or as the product of platelet division (**A**). The red-green doublets can only form by incidental contact (happen-to-be doublets) and not by division. Supposing that the platelets do not divide, the relative number of doublets of each color combination follows a binomial probability pattern. After six hours of culture, platelet singlets and doublets of each color and color combination were observed and counted using confocal microscopy (**B**). Solid arrowhead: single-colored doublet. Open arrowhead: red-green doublet.

When platelets are cultured in a viscous semisolid medium, each platelet remains in the same position without diffusive movement, as occurs in suspension culture. If a platelet divides, the two progeny platelets remain in contact with each other in the form of a doublet. Thus, if the doublets are counted and compared to the number of singlets after culture, we can estimate how many of the original platelets have divided and thus the platelet division fraction. Of course, doublets can form via incidental rendezvous of two platelets during preparation of the platelet-medium mixture (happen-to-be doublets). We argue that, if platelets are labeled with two differently colored tracking dyes (e.g., Deep Red for red and CFSE for green) and mixed together within a semisolid medium, single colored doublets could result from both division and contact, but the doublets with two colors must have formed by contact only (happen-to-be doublets) and not by division (Figure 2A). The relative number of two-colored doublets will follow a binomial probability prediction pattern if the platelets do not divide (Figure 2A). Thus, if we designate the number of red and green platelets, *R* and *G*, respectively, the relative composition of the red-red, red-green, and green-green combinations should be approximately *R*^2^/(*R* + *G*)^2^, 2*RG*/(*R* + *G*)^2^, and *G*^2^/(*R* + *G*)^2^, respectively. That composition can then be compared with the observed number of doublets of each color combination to determine whether platelet division has occurred. If there are too many single colored doublets relative to the double colored doublets far beyond a binomial prediction pattern, this can be accepted as indirect evidence of platelet division.

#### 2.4.2. Demonstration of Platelet Division by Differential Doublet Counting

Platelets were labeled with 1 μM CellTracker^TM^ Deep Red (red color: Invitrogen, Carlsbad, CA, USA) or 5 μM CFSE (green color) for 10 minutes at 37 °C. The two platelet suspensions were mixed together in a semisolid medium composed of 65% Leibowitz L-15 medium (ThermoFisher Scientific) and 35% MethoCult H4230 methylcellulose medium (STEMCELL Technologies, Vancouver, Canada). A total of 200 μL of the mixture was transferred to each culture dish for confocal microscopy imaging (35 mm; SPL, Pocheon, Korea) and incubated for six hours at 37 °C with or without treatment with 5 μM taxol (Sigma Aldrich), 5 μM nocodazole (Sigma Aldrich), or 1 μM cytochalasin D (Sigma Aldrich). The dishes were continuously maintained at 37 °C and examined on a Zeiss LSM 700 inverted confocal microscope equipped with a water immersion long working distance objective lens (63x; NA 1.15; Carl Zeiss, Jena, Germany). We counted platelet singlets and doublets of each color and color combination in ten randomly selected microscopic fields (Figure 2B). The number of triplets and other small clumps was negligible and excluded from consideration. We chose a culture time no longer than six hours because the second platelet division, possibly occurring with prolonged culture, would generate more triplets or quadruplets, making statistical prediction and testing difficult. This same culture duration was used by other researchers to show the intermediate form of dividing platelets [15,16].

## 3. Results and Discussion

### 3.1. Platelet Counting Using an Automated Hematology Analyzer Based on Coulter Counting

**Figure 3:**
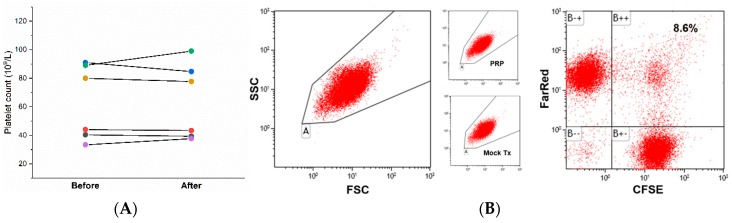
Platelet count measured by an automated Coulter counter before and after culture. The platelet counts from six blood donors before and after six hours of culture are shown by two-point series lines (**A**). The change in platelet count after culture was not significant, and the directions of change were inconsistent between the specimens. Platelet clump formation during culture was proven by flow cytometry (**B**). The platelet clumps were placed in the second (B++) quadrant, and they comprised no less than 17.2% (2 × 8.6%) of the total platelet count. The labeled platelets were the same size and shape as the unlabeled washed platelets and the untouched platelets in platelet-rich plasma (as shown in the two additional small dot plots of light scatter).

We aimed to evaluate platelet division by measuring the platelet count before and after suspension culture in a liquid medium using an automated hematology analyzer (Coulter counter). The platelet count did not change significantly after six hours of culture and the changes were not consistent among the six different blood donors (Figure 3A). The number of platelets bound to the container wall during culture and therefore not counted was difficult to assess. Platelet count can spuriously decrease secondary to platelet clump formation. Platelet clumps formed during culture can be demonstrated by flow cytometry (Figure 3B) [19]. Two platelet suspensions were prepared and labeled with 1.0 μM CellTracker^TM^ Far Red (Invitrogen) and CFSE separately. The Far Red and CFSE labeled platelets were then mixed and incubated overnight on the same rotator used for culture. The flow cytometry analysis plot shows dots in the second quadrant emitting both Far Red and CFSE fluorescence corresponding to platelet clumps (Figure 3C). These findings do not support use of the automated Coulter counter to assess platelet division.

### 3.2. Assessment of Platelet Division by CFSE Dilution Assay

**Figure 4:**
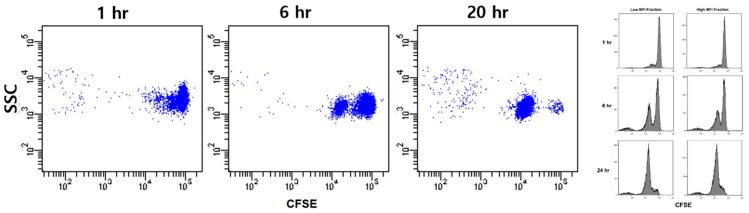
CFSE dilution assay to quantitate platelet division. Platelets were labeled with CFSE and suspended in liquid medium to be incubated for 20 hours. As with the CFSE dilution assays for lymphocytes, platelet clusters with reduced CFSE fluorescence increased progressively with culture time. The appearance of discrete clusters with reduced fluorescence indicates platelet division.

We sorted CFSE-labeled platelets such that the platelets proceeding to culture were all included in a narrow interval of fluorescence intensity. For each platelet preparation, we applied two sorting gates with one set at a high and the other at a low fluorescent region to represent large and small platelets. After six hours of culture, we observed a separate cluster of platelets with a reduced CFSE intensity adjacent to the original platelet cluster (Figure 4). As the clusters were clearly separated with no smear of dots between, the fluorescence-reduction was caused by platelet division rather than the shedding of platelet protein contents. The discrete fluorescence-reduction was the same as the typical findings from CFSE dilution assays done on lymphocytes. We continued the culture for 20 hours. The division fraction of strongly labeled platelets was 48.7% (mean, range: 27.5–70.4) after six hours and 85.6% (76.4–96.0) after 20 hours. For weakly labeled platelets, the division fraction after six hours was 35.0% (24.6–43.4) and 75.5% (34.5–97.0) after 20 hours (Table 1). There was no significant difference between platelets sorted from the high and low fluorescence regions.

### 3.3. Assessment of Platelet Division by Differential Doublet Counting

The fractions of platelet division shown by the CFSE dilution assays were somewhat higher than expected. We considered the potential effect of shear stress to which platelets were exposed during the sorting procedure. Shear stress has been shown to enhance the fission of pre-platelets and thereby increase the production of small platelets from them [16,20]. We devised a differential doublet counting method to examine platelet division in the absence of shear stress. The culture method itself using a semisolid medium was the same as that used originally to show dividing platelets.

#### 3.3.1. Demonstration of Platelet Division by Differential Doublet Counting

First, we examined whether differential doublet counting could demonstrate platelet division. After dispensing the medium containing platelets into dishes, but before proceeding to the six-hour culture, we spun down one dish from each pair and counted the platelet singlets and doublets of each color and color combination. Because there were no platelets that completed division, every doublet as observed by microscopy had to originate from incidental contact between two platelets (happen-to-be doublets). Thus, the counted numbers of red-red, red-green, and green-green doublets must follow a binomial prediction pattern at this stage. The number of each color combination was consistent with binomial prediction in each of the series of six independent experiments (*P*: 0.39–1.00, Table 2). However, after six hours of culture, the relative number of single-colored doublets (red-red, green-green) increased, and that of red-green doublets decreased far beyond the proportion expected based on binomial prediction. The same results were obtained across six independent experiments (*P* < 0.01, in the order of 10-8 to 10-5, Table 2). Because the platelets put into the culture remained in the medium, the decrease in proportion of red-green doublets was caused by an increase in the same colored doublets, i.e., platelet division.

#### 3.3.2. Cytoskeletal Rearrangement in Platelet Division

Platelet generation and division involve cytoskeletal rearrangement and reorganization in the same way as cellular division [21,22]. A change in platelet shape coupled to platelet activation also accompanies cytoskeletal rearrangement and reorganization. We treated platelets with inhibitors of microtubular disassembly or reorganization (taxol), microtubular assembly (nocodazole), or actin polymerization (cytochalasin D) throughout the six-hour culture period. The differential doublet counts followed the binomial prediction, even after six hours of culture (Ptaxol = 0.26, Pnocodazole = 0.38, and Pcytochalasin-D = 0.61, Table 3), implying that platelet division was suppressed by each of the three substances. These results mean that differential doublet counting confirms platelet division as an active biological phenomenon involving cytoskeletal rearrangement and not simple physical fission or fragmentation.

### 3.4. Derivation of the Dividing Fraction of Platelets from Differential Doublet Counting

#### 3.4.1. Derivation of the Formulae for Platelet Division Fraction

**Figure 5:**
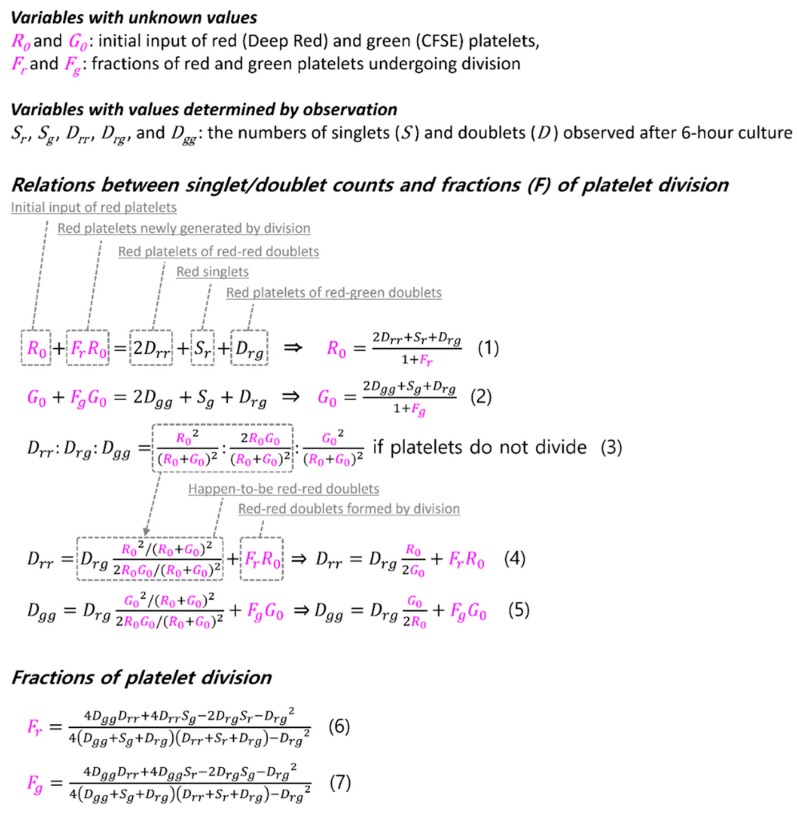
Derivation of the formulae for the platelet division fraction. The number of platelets (red or green) counted by microscopic observation equals the original input of platelets (*R*_0_ or *G*_0_) plus the newly generated platelets (platelet division: *F_r_R*_0_ or *F_g_G*_0_; Equations (1) and (2). Because the red-green doublets must have been formed by two platelets that happened to come into contact and not by division, the number of the happen-to-be red-red doublets can be calculated from their binomial coefficient relationship (Equation (3)). The observed number of red-red doublets equals the number of those happen-to-be red-red doublets plus the doublets formed by division (*F_r_R*_0_; Equation (4)). By replacing *R*_0_ and *G*_0_ in Equations (4) and (5) with those in Equations (1) and (2), the fractions of platelet division (*F_r_* and *F_g_*) can be expressed by the variables with known values. The variables with unknown values are written here in magenta.

We were able to derive a formula for the fraction of platelets undergoing division using the counted numbers of platelet singlets and doublets of the two colors. The formulation is based on two ideas (Figure 5). First, after short-term (six hours) culture, the total number of red platelets observed in the sampled microscopic fields is the sum of the numbers of red singlets, red-red doublets, and red platelets in the red-green doublets. The number of red platelets is in turn equal to the number of red platelets originally put into the sampled microscopic fields (*R*_0_) plus the red platelets newly generated by division (*F_r_* × *R*_0_), as shown by Equation (1) in Figure 5. From this equality, the original *R*_0_, which is unknown, can be expressed by the number of red-red, red-green doublets, red singlets, and the fraction of red platelet division (*F_r_*). The same reasoning can be applied to the green platelets to yield Equation (2). The second idea is that the number of doublets of the three color combinations (red-red, red-green, and green-green) should follow the binomial prediction pattern under the assumption that the platelets do not divide as argued above (Equation (3)). Because red-green doublets cannot be produced by platelet division, but only by the incidental contact of two platelets, which is a chance phenomenon, the number of happen-to-be red-red and green-green doublets can be predicted from the number of red-green doublets. The number of red-red doublets counted after six hours of culture is equal to the number of happen-to-be red-red doublets plus the red-red doublets generated by division, which is the product of *F_r_* and *R*_0_ (Equation (4)). The same reasoning is applied to yield Equation (5) for the green-green doublet count. From Equations (1), (2), (4), and (5), the dividing fractions of red and green platelets can be calculated and expressed by the numbers of singlets and doublets of each color and color combination, which we get from observation after six hours of culture (Equations (6) and (7)).

#### 3.4.2. Calculated Platelet Division Fraction 

We calculated the platelet division fraction in the experiments shown above. Table 4 summarizes the calculated division fractions and their confidence intervals. The division fractions of the Deep Red (red) platelets ranged from 9.2 to 17.5%, and those of the CFSE-labeled (green) platelets spanned from 8.8 to 17.2%. The fractions calculated before culture fell between −8.2 and 0.8% with their confidence intervals either including or below zero, indicating no division occurred. Except for two values of fractions calculated for green platelets, the confidence intervals before and after culture did not overlap, demonstrating the quantitative applicability of our method.

The differential doublet counting method sensitively detected and quantitated platelet division. The area of microscopic observation in each experiment was comparable to the scale covered by various microfluidic devices. Thus, this simple method can be modified and further evaluated for incorporation into microfluidic devices capable of assessing the division of biological particles (e.g., bacteria) that are not suitable for Coulter counting or manual counting. Of course, a sizeable live cell imaging system with a fluorescence microscope put into a cell culture-compatible space and equipped with a powerful image analysis tool can also do the same job. However, considering its simplicity and low cost, the differential doublet counting method is an attractive alternative.

## 4. Conclusions

We introduced novel methods to assess platelet division in culture conditions that can be easily applied to studies of other dividing biologic particles involving microfluidic devices. First, we found that platelet counting with automated Coulter counters is not an appropriate method to assess platelet division. However, we were able to identify and quantitate platelet division under culture conditions by modifying the CFSE dilution assay. Finally, we devised differential doublet counting as a simple way to assess platelet division. The method also needed much smaller amounts of specimen than former methods. By counting platelet doublets of different color combinations under microscopic observation, we identified platelet division and quantitated the fraction of platelets undergoing division during a six-hour culture period.

## Figures and Tables

**Table 1:** The percent of platelets undergoing division based on reduced CFSE fluorescence after sorting and culture.

Experiment Number	Initial		6 Hours		20 Hours	
	High MFI	Low MFI	High MFI	Low MFI	High MFI	Low MFI
Sort-1	15.5	9.4	41.4	43.4	90.6	97.0
Sort-2	14.3	7.9	39.7	41.8	87.3	86.5
Sort-3	10.9	6.3	27.5	27.7	81.1	34.5
Sort-4	16.4	10.9	64.5	37.7	76.4	62.4
Sort-5	12.0	11.1	70.4	24.6	83.3	48.1
Sort-6	38.5	20.3			93.8	96.5
Sort-7	15.6	11.4			89.5	70.7
Sort-8	22.1	45.3			80.4	90.0
Sort-9	15.6	11.8			60.6	68.8
Sort-10	14.3	8.0			91.7	67.5
Sort-11	12.7	3.9			95.9	92.3
Sort-12	8.2	3.5			96.0	91.3
	16.3 ± 7.8	12.5 ± 11.2	48.7 ± 18.1	35.0 ± 8.4	85.6 ± 10.1	75.5 ± 20.2
			*P* = 0.81 *		*P* = 0.15 *	

* No significant difference in division between platelets strongly (high MFI) and weakly (low MFI) labeled with CFSE.

**Table 2:** The number of platelet singlets and doublets of each color and color combination after six hours of culture in semisolid medium.

		Experiment Number (MethoCult Medium Culture)					
		MCX1	MCX2	MCX3	MCX4	MCX5	MCX6
**Initial** Doublet %* **(Expected** **^†^)**	*R* ●	67	83	407	125	114	129
	*G* ●	60	77	448	104	110	125
	*RR* ●●	3 27.3 * (27.8 ^†^)	3 21.4 * (26.9 ^†^)	11 20.0 * (22.7 ^†^)	7 21.2 * (29.8 ^†^)	4 11.1 * (25.9 ^†^)	5 14.7 * (25.8 ^†^)
	*GG* ●●	3 27.3 * (22.3 ^†^)	4 28.6 * (23.2 ^†^)	12 21.8 * (27.5 ^†^)	6 18.2 * (20.6 ^†^)	5 13.9 * (24.1 ^†^)	4 11.8 * (24.2 ^†^)
	*RG* ● ●	5 45.5 * (49.8 ^†^)	7 50.0 * (49.9 ^†^)	32 58.2 * (49.9 ^†^)	20 60.6 * (49.6 ^†^)	27 75.0 * (50.0 ^†^)	25 73.5 * (50.0 ^†^)
	*P* **^‡^**	0.39	0.50	0.89	0.90	1.00	1.00
**6-HourCulture** Doublet %* **(Expected** **^†^** **)**	*R* ●	171	124	157	132	126	122
	*G* ●	167	134	151	127	127	103
	*RR* ●●	28 48.3 (25.6 ^†^)	22 39.1 (26.0 ^†^)	18 39.1 (26.0 ^†^)	24 49.0 (26.0 ^†^)	19 44.2 (24.8 ^†^)	19 47.5 (29.4 ^†^)
	*GG* ●●	22 37.9 (24.4 ^†^)	24 41.3 (24.0 ^†^)	19 41.3 (24.0 ^†^)	16 32.7 (24.0 ^†^)	15 34.9 (25.2 ^†^)	14 35.0 (21.0 ^†^)
	*RG* ● ●	8 13.8 (50.0 ^†^)	8 19.6 (50.0 ^†^)	9 19.6 (50.0 ^†^)	9 18.4 (50.0 ^†^)	9 20.9 (50.0 ^†^)	7 17.5 (49.6 ^†^)
	*P* **^‡^**	<0.01 (1.76 × 10^−8^)	<0.01 (1.23 × 10^−7^)	<0.01 (1.85 × 10^−5^)	<0.01 (4.80 × 10^−6^)	<0.01 (6.88 × 10^−5^)	<0.01 (2.39 × 10^−5^)

* The percentage of doublets with each color combination; ^†^ The percentage of doublets with each color combination predicted by binomial probability; ^‡^ The probability of the number of red-green doublets being equal to or smaller than the observed count.

**Table 3:** Platelet division in a semisolid medium is affected by cytoskeletal reorganization involving microtubules and actin.

		Mock Treatment			Cytoskeletal Treatment			
		Count	Doublet %(Expected)		Total	Doublet %(Expected)		
**Taxol**	*R* ●	157		*P* < 0.01 ^‡^ (4.62 × 10^−5^)	164		*P* = 0.57 ^‡^	
	*G* ●	149			144			
	*RR* ●●	8	44.7 * (28.4 ^†^)		21	25.8 * (26.3 ^†^)		
	*GG* ●●	7	34.0 * (21.9 ^†^)		16	22.6 * (23.7 ^†^)		
	*RG* ● ●	16	21.3 * (49.8 ^†^)		10	51.6 * (50.0 ^†^)		
**Nocodazole**	*R* ●	149		*P* < 0.01 ^‡^ (1.97 × 10^−5^)	168		*P* = 0.67 ^‡^	
	*G* ●	146			166			
	*RR* ●●	11	37.5 * (25.3 ^†^)		15	24.4 * (25.5 ^†^)		
	*GG* ●●	10	45.0 * (24.7 ^†^)		18	22.2 * (24.5 ^†^)		
	*RG* ● ●	24	17.5 * (50.0 ^†^)		7	53.3 * (50.0 ^†^)		
**Cytochalasin-D**	*R* ●	346		*P* < 0.01 ^‡^ (3.03 × 10^−6^)	301		*P* = 0.61 ^‡^	
	*G* ●	313			281			
	*RR* ●●	15	39.6 * (26.7 ^†^)		21	26.8 * (27.6 ^†^)		
	*GG* ●●	12	41.5 * (23.3 ^†^)		22	21.4 * (22.6 ^†^)		
	*RG* ● ●	29	18.9 * (49.9 ^†^)		10	51.8 * (49.9 ^†^)		

* The percentage of doublets with each color combination; ^†^ The percentage of doublets with each color combination predicted by binomial probability; ^‡^ The probability of the number of red-green doublets being equal to or smaller than the observed count.

**Table 4:** The platelet division fractions determined from a series of six six-hour semisolid medium cultures.

	Fraction of Division	Experiment Number (MethoCult Medium Culture)					
		MCX1	MCX2	MCX3	MCX4	MCX5	MCX6
**Initial**	*F_r_* (95 % CI*)	0.8% (−5.3–6.8)	−1.0% (−6.3–4.2)	−0.8% (−2.8–1.1)	−4.0% (−9.6–1.5)	−8.2% (−13.1–-3.3)	−5.7% (−10.4–-1.1)
	*F_g_* (95 % CI*)	0.8% (−5.3–6.8)	1.1% (−4.6–7.1)	−1.3% (−3.3–0.7)	−2.0% (−7.6–3.5)	−6.6% (−11.8–-1.4)	−6.1% (−10.5–-1.8)
**6-HourCulture**	*F_r_* (95 % CI*)	16.4% (9.5–23.3)	17.3% (8.8–25.8)	9.2% (3.2–15.3)	17.5% (9.1–25.9)	13.2% (5.4–21.0)	14.0% (5.8–22.2)
	*F_g_* (95 % CI*)	12.0% (5.8–18.3)	17.2% (8.8–25.6)	10.8% (4.4–17.2)	9.7% (2.6–16.8)	8.8% (1.9–15.7)	11.8% (3.8–19.8)

* Confidence interval.
